# CMV retinitis screening and treatment in a resource-poor setting: three-year experience from a primary care HIV/AIDS programme in Myanmar

**DOI:** 10.1186/1758-2652-14-41

**Published:** 2011-08-15

**Authors:** NiNi Tun, Nikolas London, Moe Kyaw Kyaw, Frank Smithuis, Nathan Ford, Todd Margolis, W Lawrence Drew, Susan Lewallen, David Heiden

**Affiliations:** 1 Medical Action Myanmar, Yangon 11000, Myanmar; 2 Wills Eye Institute, Retina Service, Philadelphia, PA 19107, USA; 3 Médecins Sans Frontières OCA, Yangon, 11000, Myanmar; 4 Médecins Sans Frontières, London, EC1N 8QX, UK; 5 Centre for Infectious Disease Epidemiology and Research, University of Cape Town, 7925, South Africa; 6 University of California San Francisco, San Francisco, CA 94143, USA; 7 Kilimanjaro Centre for Community Ophthalmology, Moishe, Tanzania; 8 California Pacific Medical Center, San Francisco, CA 90000, USA; 9 Seva Foundation, Berkeley, CA 94710, USA

## Abstract

**Background:**

Cytomegalovirus retinitis is a neglected disease in resource-poor settings, in part because of the perceived complexity of care and because ophthalmologists are rarely accessible. In this paper, we describe a pilot programme of CMV retinitis management by non-ophthalmologists. The programme consists of systematic screening of all high-risk patients (CD4 <100 cells/mm^3^) by AIDS clinicians using indirect ophthalmoscopy, and treatment of all patients with active retinitis by intravitreal injection of ganciclovir. Prior to this programme, CMV retinitis was not routinely examined for, or treated, in Myanmar.

**Methods:**

This is a retrospective descriptive study. Between November 2006 and July 2009, 17 primary care AIDS clinicians were trained in indirect ophthalmoscopy and diagnosis of CMV retinitis; eight were also trained in intravitreal injection. Evaluation of training by a variety of methods documented high clinical competence. Systematic screening of all high-risk patients (CD4 <100 cells/mm^3^) was carried out at five separate AIDS clinics throughout Myanmar.

**Results:**

A total of 891 new patients (1782 eyes) were screened in the primary area (Yangon); the majority of patients were male (64.3%), median age was 32 years, and median CD4 cell count was 38 cells/mm^3^. CMV retinitis was diagnosed in 24% (211/891) of these patients. Bilateral disease was present in 36% of patients. Patients with active retinitis were treated with weekly intravitreal injection of ganciclovir, with patients typically receiving five to seven injections per eye. A total of 1296 injections were administered.

**Conclusions:**

A strategy of management of CMV retinitis at the primary care level is feasible in resource-poor settings. With appropriate training and support, CMV retinitis can be diagnosed and treated by AIDS clinicians (non-ophthalmologists), just like other major opportunistic infections.

## Background

In south-east Asia, cytomegalovirus (CMV) retinitis is a neglected disease [1], with no defined strategy for management [2,3]. This is despite evidence that CMV retinitis is a common cause of HIV-associated blindness in this region [4] and the second most common opportunistic infection to emerge during initiation of antiretroviral therapy (ART) [5], and that CMV viremia is a strong predictor of mortality [6].

The emerging body of data from resource-limited settings closely mirrors what was learned several decades ago in western countries about CMV infection in patients with AIDS. At that time, about one-third of patients with AIDS developed CMV retinitis, accounting for more than 90% of cases of HIV-related blindness [7,8]. Furthermore, extra-ocular CMV disease was a major cause of AIDS-related morbidity and mortality [9-11].

In resource-limited settings, the management of CMV retinitis is inadequate. Primary care clinicians have been reluctant to engage in the care of CMV retinitis, partly because of inadequate training in diagnostic approaches and partly because the most commonly available treatment - intraocular injection - has been viewed as a procedure that only an ophthalmologist could perform safely. But ophthalmologists are limited in number and often distant from the need, based in urban secondary or tertiary care facilities [12]. In addition, many ophthalmologists in developing countries may lack the skills or equipment for adequate management of CMV retinitis. The end result is that when patients are referred to ophthalmologists they commonly arrive when the disease is at a late stage and outcomes are poor. Thus, there is a need for a simple and effective system for management of CMV retinitis that can be implemented at the primary care level.

This unmet need was apparent in the Médecins Sans Frontières (MSF) AIDS project in Myanmar, a country with limited resources and the third highest prevalence of HIV in south-east Asia (adult prevalence 0.67%). In Myanmar, about 240,000 persons are living with HIV/AIDS. There are about 13,000 incident infections and 25,000 AIDS-related deaths each year [13,14]. Availability of ART in Myanmar is limited, with only about one in four people in need of ART receiving medication.

In this article, we report on a pilot programme for the integration of management of CMV retinitis into routine care for patients with HIV/AIDS at the primary care level in Myanmar.

## Methods

### Programme setting

MSF provides HIV/AIDS services and ART to more than 15,000 persons in Myanmar through 15 clinics in Yangon Division, Shan State, Kachin State and Rahkine State. Services include health education, HIV screening, counselling, treatment of opportunistic infections, nutritional support and ART.

In November 2006, a screening programme for CMV retinitis was initiated within the MSF HIV programme, with training provided by a consultant ophthalmologist to a national HIV/AIDS clinician with no prior ophthalmology training. This clinician then assumed responsibility for programme supervision and development. Selection of subsequent clinicians was primarily based on the clinical needs in the different geographic locations in Myanmar where AIDS clinics are run by MSF. Clinicians were qualified for selection if they had at least one year of experience in the AIDS clinic, were interested in learning how to manage CMV retinitis, and were judged by their supervisors to be highly motivated and with a strong commitment to clinical care. All were under 30 years of age. Formal training workshops were started in 2007, and all 17 AIDS clinicians in this programme had clinical training directly from a consultant ophthalmologist in a workshop setting.

### Screening and diagnosis

CMV screening was included as part of the protocol for clinical evaluation of all consecutively enrolled new patients in the ART programme from November 2006. As the service became known, patients were referred from private clinics, government hospitals, and non-governmental organizations.

Screening consisted of examination of the entire retina using an indirect ophthalmoscope (screening for ocular symptoms was attempted in a similar setting and found to be unreliable [1]). The pupil was fully dilated with topical neosynephrine 2.5% and tropicamide 1%. The diagnosis of CMV retinitis was based on clinical examination of the retina.

Screening criteria were broad, reflecting the principle that examination of the retina should be part of the basic physical examination of all AIDS patients at high risk for opportunistic infections. Patients who met any of the following criteria were screened: CD4 count below 100 cells/mm^3^; ocular symptoms consistent with CMV retinitis (blurred vision, floaters, scotomata, photopsia); symptoms consistent with extraocular CMV such as unexplained diarrhoea or dysphagia; symptoms of meningitis (fever, headache, altered mental status); herpes zoster ophthalmicus; and suspicion of disseminated tuberculosis. Patients with cotton-wool spots at the first screening visit were re-examined every three weeks until the spots resolved. Patients with normal retinas at the first screening visit were re-screened every three months as long as their CD4 counts remained under 100 cells/mm^3^.

### Treatment

Patients diagnosed with active CMV retinitis were treated on the same day with intraocular ganciclovir (2.5 mg ganciclovir in 0.05mL of solution) followed by weekly injections for as long as clinically required. Injections were administered in the AIDS clinic by standard procedure, using an eyelid speculum, betadine preparation, and sterile no-touch technique. If patients were not yet on ART, plans were made to initiate ART, if possible about two weeks after the first ganciclovir injection. Patients already on ART with immune reconstitution and inactive CMV retinitis were observed. Most of these patients had been treated with ART for four to six months and were referred from other programmes with visual complaints.

### Training approach

A training workshop for the diagnosis and treatment of CMV retinitis was developed and refined in Myanmar over three years. The curriculum focused narrowly on teaching indirect ophthalmoscopy and management of CMV retinitis. The training was task oriented: trainees needed to be able to identify active and inactive CMV retinitis in order to make the clinical decision to either start or discontinue CMV treatment.

The training workshop was four days long and included short didactic lectures on relevant ocular anatomy, CMV retinitis and other HIV/AIDS-related retinal pathology, as well as training in indirect ophthalmoscopy using model eyes. This was followed by case-based teaching using patients with AIDS-related eye disease. The AIDS clinicians were taught how to perform rapid bedside screening for blindness and visual field loss and palpation for low intraocular pressure secondary to retinal detachment. AIDS clinicians working in locations where intraocular injection skills were otherwise not available were carefully trained in intraocular injection of ganciclovir (intravenous ganciclovir and oral valganciclovir are not available in Myanmar). Throughout the workshop, there were case-based drills using photographic clinical material.

The goal of this curriculum was specifically to train AIDS clinicians to manage CMV retinitis, not to develop "primary eye care providers". No attempt was made to present a systematic primary eye care curriculum.

### Evaluation of training

At the end of the first workshop, the ability of five AIDS physicians to diagnose active and inactive CMV retinitis by indirect ophthalmoscopy was assessed by Kappa statistics. Thirty patients (60 eyes) were examined after appropriate consents were obtained. Examination of these patients by a consultant ophthalmologist (DH) served as the gold standard. For statistical analysis, only the right eye was used.

Training has also been consistently evaluated by a variety of informal methods that will be described.

### Ethics

The programme evaluation was based on routine clinical data, therefore ethical review and individual patient consent were not sought. All patient information was entered into a database using coded identification numbers, and no information that could reveal patient identity was collected.

## Results

Between November 2006 and July 2009, 891 new patients were screened for CMV retinitis in the Yangon Division (Table 1). The most common reason for screening was CD4 cell count <100 cells/mm^3^. The majority of patients were male (64.3%), the median age was 32 years, and the median CD4 cell count was 38 cells/mm^3^.

**Table 1:** Baseline characteristics of patients screened for CMV retinitis in Yangon

	2006*	2007	2008	2009**	Total
Number of new patients screened	55	598	164	74	891
Number of follow-up examinations	101	496	431	262	1290
% female^†^	38.5%	32.3%	41.7%	38.5%	35.7%
Median age (years) ^†^	30	32	32	33	32
Median CD4 (IQR)	32 (16-56)	36 (20-60)	44 (25-87)	41 (21-65)	38 (21-66)

† 3% missing data.

* Data collection started November 2006

** Includes data until June 2009

CMV retinitis was diagnosed in 211 of 891 (24%) new patients in Yangon Division, with bilateral disease in 76 of 211 (36%) patients. Of 1782 eyes screened, 287 (16%) were diagnosed with CMV retinitis (Table 2). CMV screening declined in 2008 and 2009, mainly due to programme resource constraints. For Shan State, Kachin State and Rahkine State, data is incomplete except for the information that an additional 268 patients were screened in Shan State and 292 in Kachin State.

**Table 2:** Diagnosis following screening for CMV retinitis in Yangon (by eyes)

	2006*	2007	2008	2009**	Total
Active CMVR	29 (26%)	93 (8%)	40 (15%)	44 (30%)	216 (12%)
Inactive CMVR	10 (9%)	17 (1%)	26 (8%)	18 (12%)	71 (4%)
No CMV	69 (63%)	1083 (91%)	232 (71%)	14 (9%)	1398 (78%)
Missing data or other	2 (2%)	3 (0.3%)	20 (6%)	72 (49%)	97 (5%)
Total	110 (100%)	1196 (100%)	328 (100%)	148 (100%)	1782 (100%)

* Data collection started November 2006

** Includes data until June 2009

Percentages may not add up to exactly 100% due to rounding.

The five physicians who participated in the first workshop were assessed with a Kappa agreement analysis for their ability to diagnose active and inactive CMV retinitis with the indirect ophthalmoscope. With 30 patients, the retina of two eyes could not be examined due to cataracts. Of the remaining 58 eyes, 29 had no disease, 23 had active CMV retinitis, and 15 had retinal scars consistent with inactive CMV retinitis (in addition, six eyes had retinal detachment, three eyes had cotton-wool spots, and six had choroidal granulomas characteristic of tuberculosis). One of the clinicians (NNT) had been instructed by the consultant ophthalmologist the previous year and already had one year of clinical experience.

The Kappa statistic for the clinician with one year experience was 1.0 (perfect agreement) for recognizing both active and inactive CMV retinitis. The strength of agreement (Kappa statistic) for the four other AIDS clinicians taking the workshop for the first time ranged from 0.73 to 0.51 (average of 0.64 or "substantial" agreement) for the diagnosis of active CMV retinitis, and 0.72 to 0.39 (average of 0.55 or "moderate" agreement) for the recognition of inactive CMV retinitis.

Workshop performance was also evaluated with tests using photographic images of retinal lesions from AIDS patients. In tests given on the final day of the three workshops during the period covered in this report, the average score of the 17 AIDS clinicians was 94%. Self-evaluations carried out on the final morning of each workshop consistently reported a high level of confidence about the ability to examine the eye with the indirect ophthalmoscope, a high level of confidence in recognizing the key retinal landmarks, and a moderate to high confidence in making the diagnosis of active and inactive CMV retinitis. The results of self-evaluation were consistent with the impression of the workshop instructor who, at the time of patient examinations, reviewed all of the retinal drawings produced by each of the AIDS clinicians as part of their workshop training.

By July 2009, 17 AIDS clinicians had been trained in the diagnosis of CMV retinitis by indirect ophthalmoscopy, eight had been trained in the intraocular injection of ganciclovir, and 1296 intraocular injections of ganciclovir already had been performed. Thirty-four injections were directly observed by the consultant ophthalmologist (DH). In the course of 1296 intraocular injections, there was a single case of infectious endophthalmitis. Minor complications (such as subconjunctival hemorrhage) were not recorded. There have been no significant complications from routine dilation of the pupil in a non-ophthalmic setting (no attacks of angle-closure glaucoma).

Four AIDS clinicians were re-evaluated one year after training. In side-by-side examination of patients who had been treated with intraocular injection for CMV retinitis by the AIDS clinicians, consultant ophthalmologists (DH, NL) confirmed the correct diagnosis of CMV retinitis in 213 of 218 eyes (98%) or 161 of 166 patients (97%). The five incorrect diagnoses were syphilis (n = 1), myelinated nerve fibre layer (n = 1), tuberculosis (n = 2), and ocular toxoplasmosis (n = 1).

## Discussion

This report documents the feasibility of training primary care AIDS clinicians to diagnose and treat CMV retinitis in a resource-limited setting. CMV retinitis screening is now carried out in four regions in Myanmar, and at the beginning of 2010, covered the majority of patients treated with ART in the country.

The high prevalence of CMV retinitis that we identified and the severity of the consequences of CMV retinitis demonstrate the importance of routine CMV retinitis screening in this setting. CMV retinitis was diagnosed in 211 out of 891 (24%) new patients screened in the Yangon Division, and these patients required urgent treatment to prevent blindness. Blindness has catastrophic consequences for these patients who are at a relatively young age, as well as for their families.

We are aware of the potential risk of causing an attack of acute angle-closure glaucoma and blindness by dilation of the pupil in a setting where back-up ophthalmic care may not be available. However, we consider that overall, the balance of risks finds in favour of using this approach: while the risk of angle closure is low (no episodes thus far), the risk of blindness from undetected and untreated CMV retinitis in this population is high.

Our experience in Myanmar demonstrates the feasibility of training AIDS clinicians to diagnose and treat CMV retinitis. Even with a limited background in ophthalmology, CMV retinitis is not difficult to diagnose. At the end of the four-day workshop, most AIDS clinicians were able to begin screening patients with the indirect ophthalmoscope, and after three to six months of practice, most were highly proficient. By one year, AIDS clinicians regarded CMV retinitis as the easiest diagnosis to establish among major opportunistic infections whereas previously, it was regarded as the most difficult. However, diagnosis of the ophthalmologic complications of CMV retinitis, retinal detachment and immune recovery uveitis (IRU) remains a challenge, as does distinguishing active from inactive retinitis in patients with IRU.

This report comes from a routine programme and as such there are several potential limitations to note. It is possible that some clinical events were missed due to missed appointments or unrecorded events. We are confident that the recording of important clinical events is reliable given that a strong emphasis is placed on data collection to support cohort monitoring in this programme. As is the case for HIV/AIDS care generally in many settings in the developing world, this programme receives support from a non-governmental organization that provides additional resources that may limit the potential for replication in settings where such additional resources are lacking. However, we consider that with adequate commitment, training and support, the approach could be extended to other, similar settings.

The set-up cost for a CMV retinitis screening programme is modest: the only equipment needed is a portable battery-operated indirect ophthalmoscope with a 28 diopter lens. Dilating drops are inexpensive. Only one clinician needs to be trained per centre, and that clinician carries out all the screening and patient management so as to remain highly practiced and skilled. Screening of one patient (two eyes) takes approximately three minutes, and one intraocular injection takes 15-20 minutes. In Myanmar, diagnostic screening and treatment has usually been managed within a single half-day clinic each week.

Treatment, however, remains problematic. As we found, treatment with intraocular injection of ganciclovir can safely be implemented at the primary care level by non-ophthalmologists, and we strongly support this treatment intervention in the absence of alternatives. Ganciclovir injection is certainly affordable [1], costing less than US$1.00 per weekly injection, and intraocular injection of ganciclovir is highly effective at controlling retinitis in the injected eye. However, intraocular injection does not treat or prevent against potentially fatal extra-ocular CMV disease, nor does it prevent the development of disease in the contralateral eye. It requires weekly clinic visits that may be cumbersome, and patients must endure repeated injections into the eye.

In contrast, patients in developed countries are treated for the same problem with a simple pill. Systemic treatment of CMV retinitis with oral valganciclovir is the standard of care in western countries [14,15]. Reduction in mortality has been observed with systemic treatment of CMV retinitis [16], even in patients failing ART therapy [17]. Although we are not able to provide outcome data in support for this standard of care in this report, we consider that that available evidence supports the use of intraocular injection as a valuable step in providing high-quality care to patients with CMV retinitis. However, intraocular injection alone is not adequate. Systemic treatment with oral valganciclovir [18] should be made affordable and widely available.

Future research should more adequately document the prevalence of CMV in resource-limited settings, and better evaluate treatment outcomes for patients treated with valganciclovir and intraocular ganciclovir, including through randomized trials.

## Conclusions

CMV retinitis, one of the major opportunistic infections of HIV/AIDS, will remain a clinical problem and cause of avoidable mortality and blindness until there is widespread early detection of HIV infection and early initiation of antiretroviral therapy at higher CD4 counts. Until that time, we believe that management of CMV retinitis needs to be integrated into routine care for patients with HIV/AIDS at the primary care level in Mynamar and similar settings, as is the done with other important opportunistic infections. Simple and effective management of CMV retinitis in resource-poor settings is a realistic goal. We recommend that other HIV/AIDS programmes in south-east Asia managing patients at potential risk of CMV retinitis move forward with similar initiatives.

## Competing interests

The authors declare that they have no competing interests.

## Authors' contributions

NNT helped design and implement the study. NJSL collected data and drafted the manuscript. MKK performed all statistical analyses and reviewed the manuscript. FS helped design and implement the study and also helped to draft the manuscript. NF reviewed and revised the manuscript. TM reviewed and revised the manuscript. WLD reviewed and revised the manuscript. SL helped implement the study and also helped draft the manuscript. DH conceived of the study, helped implement and collect data, and reviewed and revised the manuscript. All authors read and approved the final manuscript

## References

[B1] Heiden D Ford N Wilson D Rodriguez WR Margolis T Janssens B Bedelu M Tun N Goemaere E Saranchuk P Sabapathy K Smithuis F Luyirika E Drew WL Cytomegalovirus retinitis: the neglected disease of the AIDS pandemic PLoS Med 2007 14 12 e334 10.1371/journal.pmed.0040334 18052600 PMC2100142

[B2] Essential Prevention and Care Interventions for Adults and Adolescents Living with HIV in Resource-Limited Settings HIV/AIDS Programme: Strengthening health services to fight HIV/AIDS 2008 Geneva: World Health Organization Department of HIV/AIDS 1 110

[B3] Kaplan JE Benson C Holmes KH Brooks JT Pau A Masur H Guidelines for prevention and treatment of opportunistic infections in HIV-infected adults and adolescents: recommendations from CDC, the National Institutes of Health, and the HIV Medicine Association of the Infectious Diseases Society of America MMWR Recomm Rep 2009 14 RR-4 1 207 quiz CE201-204 19357635

[B4] Pathanapitoon K Ausayakhun S Kunavisarut P Wattananikorn S Leeungurastien T Yodprom R Narongjunchai D Rothova A Blindness and low vision in a tertiary ophthalmologic center in Thailand: the importance of cytomegalovirus retinitis Retina 2007 14 5 635 640 10.1097/01.iae.0000249575.38830.45 17558328

[B5] Manosuthi W Chaovavanich A Tansuphaswadikul S Prasithsirikul W Inthong Y Chottanapund S Sittibusaya C Moolasart V Termvises P Sungkanuparph S Incidence and risk factors of major opportunistic infections after initiation of antiretroviral therapy among advanced HIV-infected patients in a resource-limited setting J Infect 2007 14 5 464 469 10.1016/j.jinf.2007.07.002 17714788

[B6] Micol R Buchy P Guerrier G Duong V Ferradini L Dousset JP Guerin PJ Balkan S Galimand J Chanroeun H Lortholary O Rouzioux C Fontanet A Leruez-Ville M Prevalence, risk factors, and impact on outcome of cytomegalovirus replication in serum of Cambodian HIV-infected patients (2004-2007) J Acquir Immune Defic Syndr 2009 14 4 486 491 10.1097/QAI.0b013e3181a254c2 19421071

[B7] Holbrook JT Jabs DA Weinberg DV Lewis RA Davis MD Friedberg D Visual loss in patients with cytomegalovirus retinitis and acquired immunodeficiency syndrome before widespread availability of highly active antiretroviral therapy Arch Ophthalmol 2003 14 1 99 107 12523893 10.1001/archopht.121.1.99

[B8] Spector SA Weingeist T Pollard RB Dieterich DT Samo T Benson CA Busch DF Freeman WR Montague P Kaplan HJ A randomized, controlled study of intravenous ganciclovir therapy for cytomegalovirus peripheral retinitis in patients with AIDS. AIDS Clinical Trials Group and Cytomegalovirus Cooperative Study Group J Infect Dis 1993 14 3 557 563 10.1093/infdis/168.3.557 8394858

[B9] Yust I Fox Z Burke M Johnson A Turner D Mocroft A Katlama C Ledergerber B Reiss P Kirk O Retinal and extraocular cytomegalovirus end-organ disease in HIV-infected patients in Europe: a EuroSIDA study, 1994-2001 Eur J Clin Microbiol Infect Dis 2004 14 7 550 559 15232720 10.1007/s10096-004-1160-2

[B10] Ansari NA Kombe AH Kenyon TA Hone NM Tappero JW Nyirenda ST Binkin NJ Lucas SB Pathology and causes of death in a group of 128 predominantly HIV-positive patients in Botswana, 1997-1998 Int J Tuberc Lung Dis 2002 14 1 55 63 11931402

[B11] Pecorella I Ciardi A Credendino A Marasco A Di Tondo U Scaravilli F Ocular, cerebral and systemic interrelationships of cytomegalovirus infection in a post-mortem study of AIDS patients Eye (Lond) 1999 14 Pt 6 781 785 10707145 10.1038/eye.1999.228

[B12] Sommer A Global access to eye care Arch Ophthalmol 2007 14 3 399 400 10.1001/archopht.125.3.399 17353413

[B13] WHO/UNAIDS/UNICEF Epidemiological Fact Sheet on HIV and AIDS: Core Data on Epidemiology and Response, Myanmar 2008 Geneva: UNAIDS/WHO

[B14] Drew WL Erlich KS Volberding P, Sande M, Lange J, Greene W, Gallant J Management of virus infections (cytomegalovirus, herpes simplex virus, varicella-zoster virus) Global HIV/AIDS medicine 2007 New York: Elsevier 437 461

[B15] Jacobson MA Treatment of cytomegalovirus retinitis in patients with the acquired immunodeficiency syndrome N Engl J Med 1997 14 2 105 114 10.1056/NEJM199707103370207 9211681

[B16] Binquet C Saillour F Bernard N Rougier MB Leger F Bonnal F Dabis F Prognostic factors of survival of HIV-infected patients with cytomegalovirus disease: Aquitaine Cohort, 1986-1997. Groupe d'Epidemiologie Clinique du SIDA en Aquitaine (GECSA) Eur J Epidemiol 2000 14 5 425 432 10.1023/A:1007627508918 10997829 PMC4710783

[B17] Kempen JH Jabs DA Wilson LA Dunn JP West SK Tonascia J Mortality risk for patients with cytomegalovirus retinitis and acquired immune deficiency syndrome Clin Infect Dis 2003 14 10 1365 1373 10.1086/379077 14583871

[B18] Martin DF Sierra-Madero J Walmsley S Wolitz RA Macey K Georgiou P Robinson CA Stempien MJ A controlled trial of valganciclovir as induction therapy for cytomegalovirus retinitis N Engl J Med 2002 14 15 1119 1126 10.1056/NEJMoa011759 11948271

